# Efficacy of non-surgical interventions for promoting improved functional outcomes following acute compartment syndrome: A systematic review

**DOI:** 10.1371/journal.pone.0274132

**Published:** 2022-09-09

**Authors:** Naveena B. Janakiram, Jessica M. Motherwell, Stephen M. Goldman, Christopher L. Dearth

**Affiliations:** 1 DoD-VA Extremity Trauma and Amputation Center of Excellence, Bethesda, MD, United States of America; 2 Department of Surgery, Uniformed Services University of the Health Sciences and Walter Reed National Military Medical Center, Bethesda, MD, United States of America; Mayo Clinic Minnesota, UNITED STATES

## Abstract

**Background:**

Acute compartment syndrome (ACS) is a devastating complication which develops following a traumatic extremity injury that results in increased pressure within osteofascial compartments, thereby leading to ischemia, muscle and nerve necrosis, and creates a life-threatening condition if left untreated. Fasciotomy is the only available standard surgical intervention for ACS. Following fasciotomy the affected extremity is plagued by prolonged impairments in function. As such, an unmet clinical need exists for adjunct, non-surgical therapies which can facilitate accelerated functional recovery following ACS. Thus, the purpose of this systematic review was to examine the state of the literature for non-surgical interventions that aim to improve muscle contractile functional recovery of the affected limb following ACS.

**Methods:**

English language manuscripts which evaluated non-surgical interventions for ACS, namely those which evaluated the function of the affected extremity, were identified as per PRISMA protocols via searches within three databases from inception to February 2022. Qualitative narrative data synthesis was performed including: study characteristics, type of interventions, quality, and outcomes. Risk of bias (RoB) was assessed using the Systematic Review Centre for Laboratory Animal Experimentation’s (SYRCLE) RoB tool and reported level of evidence for each article.

**Results:**

Upon review of all initially identified reports, 29 studies were found to be eligible and included. 23 distinct non-surgical interventions were found to facilitate improved muscle contractile function following ACS. Out of 29 studies, 15 studies which evaluated chemical and biological interventions, showed large effect sizes for muscle function improvement.

**Conclusions:**

This systematic review demonstrated that the majority of identified non-surgical interventions facilitated an improvement in muscle contractile function following pathological conditions of ACS.

## Introduction

The majority of combat-related injuries within the military population are in the lower extremities [1–3]. Acute compartment syndrome (ACS) within the extremities is a devastating condition that can develop in conjunction with long bone fractures, crush injury, gunshot wounds, burns, contusions, over-exertion, or prolonged limb compressions [4, 5]. ACS is also seen in civilian populations who are involved in fire arm assaults, motorcycle crash, and pedestrian struck by automobile injuries [6]. ACS occurs when the pressure within a closed compartment space surpasses a critical threshold (perfusion pressure of 30 mmHg or less) and compromises circulation/perfusion subsequently leading to ischemia [7–10]. At the biochemical level, reactive oxygen species (ROS) generated within the ischemic tissues activate a neutrophil response via arachidonic acid inflammatory pathways [11] that ultimately leads to intracellular swelling/edema, vascular and nerve damage, and deep muscle pain at the affected tissue site. Moreover, this inflammatory response further accelerates the release of cytotoxic enzymes and excess ROS whereby an additional cascade of cellular damage is triggered. Under these conditions, skeletal muscle, specifically Type II muscle fibers, are susceptible to severe damage leading to myonecrosis and a loss of function with a prolonged/delayed recovery [12]. As such, while loss of function presents a threat of limb loss, release of cellular contents from myonecrosis in conjunction with complement activation, and pro-inflammatory cytokines accelerate the systemic inflammation which can lead to remote organ damage [13–15] and contribute to multi-organ failure, or even death if left untreated [11, 16–20]. Therefore, it is essential to relieve pressure, reduce ischemia, and restore blood circulation to salvage ischemic tissues shortly after diagnosis to limit the primary and secondary impacts of the condition.

Fasciotomy is the only standard surgical intervention available to treat ACS. Surgical treatment of ACS by fasciotomy restores perfusion within the injured compartment with immediate relief; however, in-so-doing, this procedure further contributes to an additional reperfusion injury due to the release of pro-inflammatory immune cells and by-products of muscle tissue damage [7] and a further increase in oxidative stress. In other words, reperfusion injury is multifactorial and causes extensive tissue destruction involving generation of ROS, calcium overload, capillary leakage/endothelial dysfunction, the opening of the mitochondrial permeability transition pore, thus impairing ATP production, activation of inflammatory and pro-thrombogenic cascades, mechanically and metabolically induced cytoskeletal damage. Ultimately, ACS and the associated ischemia reperfusion (I/R) injury elicits a deleterious impact on the muscle contractile functionality of the affected limb. There exists an opportunity for the development of adjunct, non-surgical therapeutics which aim to facilitate improved functional outcomes by increasing the rate of healing and reducing the overall recovery time for those afflicted with ACS.

Given the lack of clinically available non-surgical treatments for ACS, it is imperative to better understand the existing literature, across the translational research continuum, to this end. Therefore, the purpose of this study was to perform a systematic review of the ACS literature to identify all non-surgical therapeutics which aim to promote improved functional outcomes that have been investigated as a means of providing a reference for subsequent use in the development of next generation technologies. While the initial literature search conducted to this end resulted in no articles recapitulating trauma-induced ACS with outcomes on muscle function in extremities, evaluation of articles which examined interventions on tissue I/R injuries were used as a means for identifying therapeutic candidates which might address the primary aspects of the pathophysiology of ACS in extremity trauma.

## Methods

### Search strategy

A systematic review of the literature was conducted in accordance with the Preferred Reporting Items for Systematic review and Meta-Analysis (PRISMA) protocols [21]. This systematic review was not registered and a review protocol paper was not submitted. A comprehensive search of several databases (PubMed, Web of Science and Embase) was conducted by one author (N.B.J) from inception to Feb 3^rd^, 2022 using the following key word strategy: ("Trauma" OR "penetrating" OR "injury" OR "wound") AND (“extremity” OR “limb” OR “arm” OR “leg”) AND ("acute compartment syndrome" OR "compartment syndrome" OR "crush syndrome" OR "intracompartmental pressures" AND "edema" OR "edema" OR "ischemia" OR "ischemia" OR "reperfusion") AND ("therapy" OR "drug" OR "intervention" OR "treatment" OR "pharmacology" OR "medicine" OR “therapeutic” OR “small molecule” OR “biologic”) AND (“muscle function” OR "muscle strength" OR "neuromuscular strength").

### Selection criteria

Covidence software was used for primary screening and data extraction. The articles were reviewed in an un-blinded, standardized method. Two independent reviewers (NBJ and JMM) reviewed the title and abstract of all studies for eligibility and subsequently screened the full texts of those included. Discrepancies were resolved by a third reviewer (SMG). To be included in the analyses, each article was screened for all of the following criteria: 1) original studies containing primary data about non-surgical interventions for traumatic ACS of extremities, 2) utilization of a skeletal muscle functional outcome measure, 3) availability of the full text of the article, 4) reported sample size, measured variance as standard deviation (SD) of the mean or standard error of the mean (SEM) 5) reporting of the type of intervention, and 6) duration of treatment. The following exclusion criteria were applied: 1) non-traumatic, chronic, exertional, and/or pathological forms of compartment syndrome, vascular ACS and abdominal ACS, 2) studies including patients below 18 years of age, 3) biomechanical studies, ex-vivo/in-vitro studies, 4) non-primary data reports (e.g., technical reports, reviews, commentaries, conference abstracts and case-reports), 5) studies in languages other than English, 6) studies with no functional analysis of the affected musculature, and 7) non-traumatic healthy patients study.

## Extraction of data

### Clinical studies

No eligible clinical studies were found.

### Preclinical studies

The following details from included studies were extracted by two independent reviewers (NBJ and JMM): 1) publication year and the name of the first author, 2) the characteristics of the *in-vivo* model including sample size, species, sex, weight, and age, 3) anesthesia methods 4) symptoms such as edema, inflammation, and loss of function, 5) information related to treatment group(s), including type of therapeutic agent (e.g., biological or chemical), therapeutic dosage, method of administration, duration of treatment, and therapeutic agent alone or combined with another material and the same information of control group, 6) types of outcome measures evaluated, such as inflammation, edema, and muscle strength/function, and 7) group sizes, mean value and SD or SEM of outcomes. If outcomes were performed at different time points, only data from the final test was extracted for use herein. If the experimental group of animals received various doses of the drug therapy, all the data of various doses of the drug was extracted for use herein. Muscle function data was extracted from each experimental and control group of every study. Data was extracted from published figures (for unreported actual data values) using Web Plot Digitizer, version 4.5 (https://automeris.io/WebPlotDigitizer). The effect size with 95% CI was for the muscle functional outcome measure was calculated for each individual study.

### Study quality and risk of bias of the studies

Two independent reviewers (NBJ and JMM) utilized the Systematic Review Centre for Laboratory Animal Experimentation’s (SYRCLE) risk of bias (RoB) tool to assess the risk of bias [22] via the following criteria: 1) peer reviewed publication, 2) control of temperature and lighting for animal housing, 3) random allocation of animals to treatment or control groups, 4) timing of disease induction before/after randomization (ischemia/trauma), 5) random selection of animals for outcome assessment, 6) blinded assessment of outcome, 7) use of anesthetic without significant effects on the skeletal muscle injury/force production, 8) animal model (aged, young, sex, weight), 9) sample size calculation, 10) free of selective outcome reporting (i.e., if all the pre-specified primary and secondary outcomes are reported), 11) compliance with animal welfare regulations, and 12) statement of potential conflict of interests. Study quality is rated as yes scored 1, and studies rated as no or unsure scored 0. Each study quality was given overall quality of evidence scores. Lower total scores indicate a higher risk of bias.

### Data analysis and synthesis

A qualitative narrative data synthesis of included studies was conducted. Study characteristics, type of interventions, quality, and outcomes were reported according to a standard format and similarities and differences compared across studies.

## Results

### 1. Study selection

**Fig 1:**
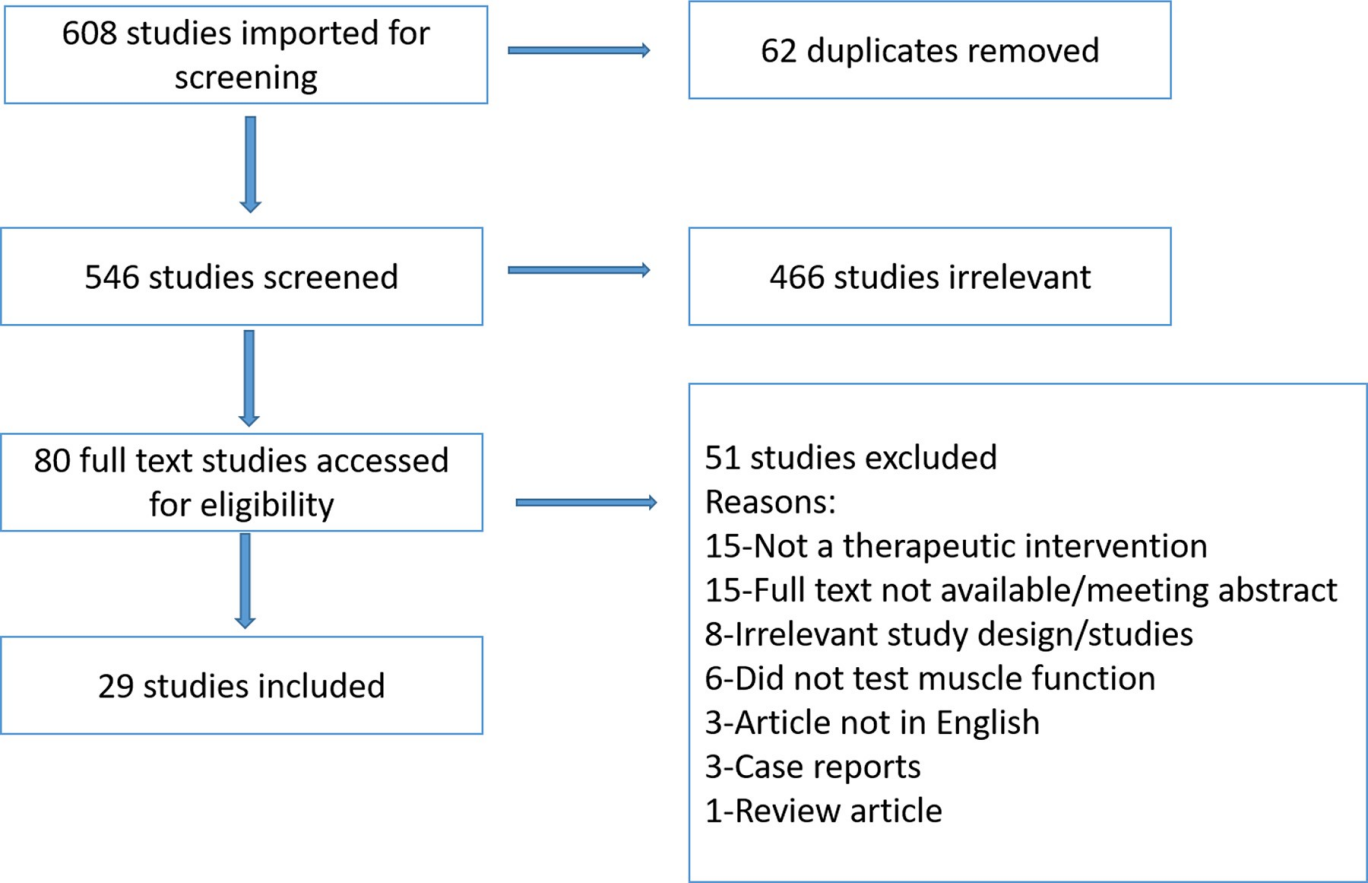
Prisma flow diagram.

Database searches yielded 608 titles with potential relevance (**Fig 1**). After excluding 62 duplicate studies, 546 studies were screened for titles and abstracts, of which 466 studies did not meet the inclusion criteria leaving 80 studies eligible for full text review (**Fig 1**). After reviewing the full text, an additional 51 studies were excluded due to the following reasons: no therapeutic investigated (n = 15), no full text available (n = 15), irrelevant study design/studies (e.g. prophylactic or other forms of compartments syndrome that are not traumatic ACS of the extremities) (n = 8), no evaluation of muscle function (n = 6), article not in English (n = 3), case report (n = 3), or a review article (n = 1). Subsequently, a total of 29 studies met the criteria for inclusion (See **Table 1**).

**Table 1:** Animal model, experimental timelines and parameters of therapeutic strategy.

First Author	Year of publication	Species	Sex	I/R method	Anesthesia	Type of intervention	Name of intervention	Route of administration	Duration of treatment	Termination of experiment	Ref
Aurora, A.	2018	Sprague-Dawley-Rat	Male	Pneumatic tourniquet	Isoflurane	Biological (fresh whole blood (FWB)	FWB- (Group- TK and HEM with FWB resuscitation)	Intravenous infusion	60 mins	28 days	[23]
Bagis, Z.	2018	Wistar albino-Rat	Male	Elastic non-pneumatic tourniquet	Ketamin hydrochloride and xylazin hydrochloride	Synthetic chemical	IL- iloprost	Intraperitoneal infusion	10 mins infusion	After 4 and 8 hrs	[32]
Chen, L. E.	1998	Sprague-Dawley-Rat	Male	Atraumatic clamps	Nembutal	Synthetic chemical	S-nitroso-N-acetylcysteine (SNAC)	Intravenous infusion	6.5 hrs	after 6.5 hrs	[24]
Chen, L. E.	1996	Sprague-Dawley -Rat	Male	Atraumatic clamps	Nembutal	Synthetic chemical	Dexamethasone	Intramuscular	One time injection	after 27 hrs	[25]
Chen, X. K	2011	Lewis -Rat	Male	Pneumatic tourniquet	Isoflurane	Biological	Muscle progenitor cells- infected with lentiviruses	Intramuscular	One time injection	After 14 days	[33]
Corona, B. T.	2014	FVB-Mouse	Female	Pneumatic tourniquet	Isoflurane	Biological	Lin-BMCs	Intravenous	One time injection	after 16 days	[41]
Corona, B. T.	2013	FVB-Mouse	No mention	Pneumatic tourniquet	Isoflurane	Biological	Lin-BMCs	Intramuscular	One time injection	After 16 days	[42]
Demirel, M	2013	Fisher -Rat	Male	Elastic non-pneumatic tourniquet	Ketamine and Xylazine	Chemical	L-carnitine	Intraperitoneal	Daily-7 days	After 7 days	[40]
Dillon, J. P.	2008	Sprague-Dawley-Rat	Male	Elastic non-pneumatic tourniquet	Thiopentone Sodium	Chemical	Hypertonic saline (7.5% NaCl)	Intravenous	15 minutes	14.5 hrs	[26]
Dillon, J. P.	2006	Sprague—Dawley-Rat	Male	Elastic non-pneumatic tourniquet	Thiopentone Sodium	Chemical	Pravastatin	Gavage	Daily-5 days	5 days	[27]
Dyer, S.E.	2022	Lewis Rat	Male	Pneumatic tourniquet	Isoflurane	Chemical and biological	Sodium percarbonate and catalase	Intramuscular	One time injection	28 days	[34]
Feller, A. M	1989	NZ-Rabbits	Male	Atraumatic clamps	Ketamine hcl and acepromazine maleate and general anesthesia by 1% halothane, 50% oxygen and 49% nitrous oxide by mask.	Biological and chemical	SOD & DMSO	Intravenous	One time injection	After 5 and 8 hrs	[48]
Frey, S. P.	2019	NZ-Rabbits	No mention	Atraumatic clamps	General Anesthesia	Biological	CYR61	collagen matrix coated with CYR61- placed at site of osteotomy	One time injection	40 days	[51]
Hirose, J.	1997	Lewis Rat	Male	Amputated the thigh sparing femoral vessels, later femoral vessels were occluded to avoid venous congestion	Sodium Pentobarbital	Synthetic chemical	L-ascorbic acid 2-[3,4-dihydro-2,5,7,8-tetrameth- ~1-2-(4,8,12-trimethyltridecy1)-2H- 1 -benzopyran-6- yl hydrogen phosphate] potassium salt (EPC-K1)	Intravenous	One time injection	24 hrs	[35]
Hirose, J.	2001	Lewis Rat	Male	Amputated the thigh sparing femoral vessels, later femoral vessels were occluded to avoid venous congestion	Sodium Pentobarbital and Pentobarbital	Chemical	L-ascorbic acid 2-[3,4-dihydro-2,5,7,8-tetramethyl-2-(4,8,12- trimethyltridecyl)-2H-1-benzopyran-6-yl hydrogen phosphate] potassium salt (EPC-K1)	Intravenous	One time injection	24 hrs	[36]
Hoch, J. R	1991	Canine	Female	Atraumatic clamps	Acepromazine, Atropine, and Halothane	Biological and chemical	Superoxide dismutase and mannitol	intra-arterially+infusion	One time bolus +60 min infusion	1 hr	[47]
Ikebe, K.	2001	Lewis Rat	Male	Amputated the thigh sparing femoral vessels, later femoral vessels were occluded to avoid venous congestion	Sodium Pentobarbital	Chemical	L-NMMA	Intravenous infusion	2 hrs	After 24 hrs	[37]
Ikebe, K.	2002	Lewis Rat	Male	Amputated the thigh sparing femoral vessels, later femoral vessels were occluded to avoid venous congestion	Sodium Pentobarbital	Chemical	L-NMMA; L-NMMA + S-nitrosoglutathione	Intravenous infusion	2 hrs	After 24 hrs	[38]
Kingston, R.	2005	Sprague- Dawley Rat	Male	Atraumatic clamps	Isoflurane	Chemical	Taurine	Intravenous	One time injection	After 4 hrs 30 mins	[28]
Mohler, L. R.	1996	NZW-Rabbit	Male	Elastic non-pneumatic tourniquet	Ketamine + Xylazine + Acepromazine	Chemical	U74006F (tirilazad mesylate, 21-[4-(2,6-di-1-pyrrolidinyl-4-pyrimidinyl)-1-piperazinyl]16m-methyl-pregna-1,4,9(11)-triene-3,20 dione methane-sulfonate	Intravenous	3 doses every 2 hrs	2 days	[49]
Morgan, R. A	1993	DOG/Canines	Female	Pneumatic tourniquet	Sodium Pentobarbital	Chemical	ATP/MgCl2	Intravenous-infusion	3 hrs	7 hrs	[50]
Pekoglu, E	2020	Sprague-Dawley Rat	Male	Elastic non-pneumatic tourniquet	Ketamine HCL + Xylazine	Synthetic chemical	trans-cinnamaldehyde (TCA)	Gavage	3 times with an 8 hr interval	After 24 hrs	[29]
Quinones-Baldrich, W. J.	1991	NZW-Rabbits	Male	Atraumatic clamps	Ketamine and Thorazine	Pump Controlled reperfusion	Controlled reperfusion	Intravenous	30 mins	7 hrs	[46]
Ricles, L. M.	2016	Lewis Rat	Male	Ligation methods	Isoflurane infused with oxygen	Biological	MSCs + PEGylated fibrin gel	Intramuscular	One time injection	After 7 and 14 days	[39]
Rybalko, V.	2015	C57BL/6 Mouse	F &M	Pneumatic tourniquet	Isoflurane	Biological	in vitro polarized M1macrophages	Intramuscular	One time injection	after 14 days	[43]
Rybalko, V.	2017	C57BL/6 Mouse	Female	Ligation methods	Isoflurane	Biological	mMPs	Intramuscular	One time injection	Ater 21 days	[44]
Stahl, D.	2012	Sprague-Dawley Rat	Male	Ligation methods	Urethane	Temperature effects and L-arginine	L-arginine at Warm I/R	NA	0.5-mL increments over 5-minute intervals for 1 hr	After 2 hrs	[30]
Wang, Y.	2021	C57BL/6 Mouse		Ligation methods	Isoflurane	Biological (Exosomes)	NC-Exo, or si-cPWWP2A (si-Exo)	Intramuscular	One time injection	28 days	[45]
Wilson, J. S.	2003	Sprague-Dawley Rat	Female	Pneumatic tourniquet application	Ketamine and Xylazine	Chemical	Dichloroacetate (DCA)	Intravenous	One time injection	After 1 hr	[31]

### 2. Study characteristics

#### General information

All 29 articles used preclinical animals for experimentation. All studies had an appropriate control arm for the intervention used. 18 studies used rats in their experimental design. Of these 18 studies, nine articles used Sprague-Dawley rats [23–31], one used Wistar albino rats [32], seven studies used Lewis rats [33–39], and one study used Fisher rats [40]. Five of the 29 articles used mice in their experimental design. Of these five studies, two studies used Friend Virus B (FVB) mice [41, 42] and three studies used C57BL/6 mice [43–45]. Three of the 29 articles used New Zealand white rabbits [46] and one article used dogs in their experimental designs [47]. 21 studies used male animals [23–30, 32–40, 45, 46, 48, 49], five studies used female animals [31, 41, 44, 47, 50], and one study used animals of both sexes [43]. Two studies did not report the gender of the animals [42, 51] (See **Table 1**).

#### Animal model

Ischemia and I/R models were used for all the included studies (**Table 1**). These studies used either elastic non-pneumatic tourniquet [26, 27, 29, 32, 40, 49], pneumatic tourniquet [23, 31, 33, 34, 41–43], atraumatic clamps [24, 25, 28, 46–48, 51], or ligation methods [30, 39, 44, 45] to produce ischemia in the hind limbs. Specifically, four of the studies amputated the thigh sparing femoral vessels, later femoral vessels were occluded to avoid venous congestion and create ischemia [35–38]. One study clamped only the femoral artery sparing femoral vein [47]. One study used combined hemorrhage (HEM) and tourniquet -induced limb I/R.

To induce anesthesia, ten studies used isoflurane [23, 28, 33, 34, 39, 41–45], seven studies used nembutal [24, 25, 35–38, 50], four studies used ketamine and xylazine [29, 31, 32, 40], two studies from the same group used thiopentone sodium [26, 27], one study used ketamine, xylazine, and acepromazine [49], one study used ketamine and thorazine [46], one study used ketamine, acepromazine maleate, and general anesthesia by 1% halothane, 50% oxygen, and 49% nitrous oxide by mask [48], one study used acepromazine, atropine, and halothane [47], one study used unspecified general anesthesia [51], and the remaining one study used urethane [30] (see **Table 1**).

#### Experimental timelines and parameters of therapeutic strategy

Tables 1 and 2 examines a wide range of interventions including chemicals, biologicals, and others factors such as temperature effects and controlled reperfusions. Out of 29 studies, 16 used chemical interventions [24–29, 31, 32, 34–38, 40, 49, 50], nine used biological interventions [23, 33, 39, 41–45, 51], three tested both biological and chemical interventions [34, 47, 48], one study tested temperature effects and also arginine chemical [30], and one tested the effect of controlled reperfusions [46]. Chemical interventions included: iloprost-(IL), S-nitroso-N-acetylcysteine (SNAC), Dexamethasone, L-carnitine, Hypertonic saline (7.5% NaCl), Pravastatin, Sodium percarbonate, Superoxide dismutase (SOD), L-ascorbic acid 2-[3,4-dihydro-2,5,7,8-tetramethyl-2-(4,8,12- trimethyltridecyl)-2H-1-benzopyran-6-yl hydrogen phosphate] potassium salt (EPC-K1), L-NMMA, L-NMMA + S-nitrosoglutathione, Taurine, U74006F (tirilazad mesylate,21-[4-(2,6-di-1-pyrrolidinyl-4-pyrimidinyl)-1-piperazinyl]16m-methyl-pregna-,4,9(11)-triene-3,20 dione methane-sulfonate, ATP/MgCl2, trans-cinnamaldehyde (TCA), Dichloroacetate (DCA), L-arginine, and mannitol. Biological interventions included: CYR61, bone marrow-derived mesenchymal stem cells (MSCs) in PEGylated fibrin hydrogel, LPS/IFN-γ polarized macrophages, Lin-BMCs, catalase, mMPs (IFN-gamma polarized mouse bone marrow macrophages), superoxide dismutase, fresh whole blood (FWB), muscle progenitor cells infected with lentiviruses, IFN-gamma polarized mouse bone marrow macrophages and exosomes. Physical interventions used included: warm I/R and pump controlled reperfusions.

**Table 2:** Details on inflammation, edema and muscle function test.

General details			Inflammation		Oedema					Isometric Torque			
First Author	Year of publication	Name of intervention	Inflammation	Method used to test inflammation	Oedema	Time point oedema analysed	Method used to test Oedema	% difference-Control Vs Treated	P value	Muscle tested	Muscle function method	Isometric torque tested at Hz	Ref
Aurora, A.	2018	FWB-(Group- TK and HEM with FWB resuscitation)	Tested-No effect	Histology (hematoxylin and eosin stain)(neutrophils, lymphocytes, mac-rophages)	Tested- FWB had no effect and HEX incresaed oedema when compared to control.	At day 2	wet-to-dry weight ratio	Hex-12.4904% increase; FWB-3.55412% inrcease	ns and P≤0.05	TA	*In-vivo*	150	[23]
Bagis, Z.	2018	IL- iloprost	Did not test	NA	Did not test	NA	NA	NA	NA	EDL	Ex-vivo	150	[32]
Chen, L. E.	1998	S-nitroso-N-acetylcysteine (SNAC)	Tested-No efffect- inflammation was present in both control and treatment groups	Histology (hematoxylin and eosin stain-microscopic observation)	Did not test	NA	NA	NA	NA	EDL	Ex-vivo	120	[24]
Chen, L. E.	1996	Dexamethasone	Tested- Inhibited in treatment group	Histology (hematoxylin and eosin stain) Neutrophils	Did not test	NA	NA	NA	NA	EDL	Ex-vivo	100	[25]
Chen, X. K	2011	Muscle progenitor cells- infected with lentiviruses	Tested -Inhibited in treatment group	CD68 immunoreactivity, indicative of macrophage infiltration-IHF	Did not test	NA	NA	NA	NA	TA	In situ	150	[33]
Corona, B. T.	2014	Lin-BMCs	Did not test	NA	Did not test	NA	NA	NA	NA	Anterior crural muscle (i.e., TA and EDL muscles)	*In-vivo*	200	[41]
Corona, B. T.	2013	Lin-BMCs	Did not test	NA	Did not test	NA	NA	NA	NA	Anterior crural muscle (i.e., TA and EDL muscles)	*In-vivo*		[42]
Demirel, M	2013	L-carnitine	Did not test	NA	Did not test	NA	NA	NA	NA	EDL and SOL	Ex-vivo	150	[40]
Dillon, J. P.	2008	Hypertonic saline (7.5% NaCl)	Tested -Inhibited in treatment group	Myeloperoxidase activity—neutrophil infiltration	Tested- Inhibited in treatment group	14.5 hrs	Wet-to-dry ratios	180.284% decrease	P<0.01	TA	In situ	50	[26]
Dillon, J. P.	2006	Pravastatin	Tested -Inhibited in treatment group	Myeloperoxidase (MPO) activity—neutrophil infiltration.	Tested- Inhibited in treatment group	14.5 hrs	Wet-to-dry ratios	5.29986% decrease	P<0.04	Gastrocnemius muscle	In situ	50	[27]
Dyer, S.E.	2022	Sodium percarbonate and catalase	Did not test	NA	NA	NA	NA	NA	NA	TA	*In-vivo*	150	[34]
Feller, A. M	1989	SOD	Tested-No effect	Macrophage infiltration-Histology-Trichrome stain	Did not test	NA	NA	NA	NA	TA	In situ	No mention	[48]
Frey, S. P.	2019	CYR61	Did not test	NA	Did not test	NA	NA	NA	NA	TA	*In-vivo*	No mention	[51]
Hirose, J.	1997	L-ascorbic acid 2-[3,4-dihydro-2,5,7,8-tetrameth- ~1-2-(4,8,12-trimethyltridecy1)-2H- 1 -benzopyran-6- yl hydrogen phosphate] potassium salt (EPC-K1)	Tested -Inhibited in treatment group	Histology-Stained with hema- toxylin and eosin (HE) cellular inflammation-no specifics on type of cells.	Tested- Inhibited in treatment group	24 hrs	Wet-to-dry ratios	10.9989% decrease	P = 0.01	Gastrocnemius muscle	In situ	200	[35]
Hirose, J.	2001	L-ascorbic acid 2-[3,4-dihydro-2,5,7,8-tetramethyl-2-(4,8,12- trimethyltridecyl)-2H-1-benzopyran-6-yl hydrogen phosphate] potassium salt (EPC-K1)	Tested -Inhibited in treatment group	Histology-Neutrophil infiltration- stained with hematoxylin and eosin (HE).	Tested- Inhibited in treatment group	24 hrs	Wet-to-dry ratios and also by histology	8.20046% decrease	P = 0.003	Gastrocnemius muscle	In situ	200Hz	[36]
Hoch, J. R	1991	Superoxide dismutase and mannitol	Did not test	NA	Tested- Inhibited in treatment groups	1 hr	Muscle was removed and weighed and percentage weight change in the experimental muscle was calcultaed.	Superoxide dismutase-12.2449% decrease; mannitol-56.3945% decrease	Superoxide dismutase- P<0.04; Mannitol- P<0.003	TA	As previously reported	No mention	[47]
Ikebe, K.	2001	L-NMMA	Did not test	NA	Did not test	NA	NA	NA	NA	Gastrocnemius muscle and TA	In situ	150	[37]
Ikebe, K.	2002	L-NMMA; L-NMMA + S-nitrosoglutathione; Super oxide dismutase (SOD)	Did not test	NA	Did not test	NA	NA	NA	NA	Gastrocnemius muscle	In situ	200	[38]
Kingston, R.	2005	Taurine	Did not test	NA	Did not test	NA	NA	NA	NA	Gastrocnemius muscle	Ex-vivo	No mention	[28]
Mohler, L. R.	1996	U74006F (tirilazad mesylate, 21-[4-(2,6-di-1-pyrrolidinyl-4-pyrimidinyl)-1-piperazinyl]16m-methyl-pregna-1,4,9(11)-triene-3,20 dione methane-sulfonate	Did not test	NA	Did not test	NA	NA	NA	NA	TA	In situ	100	[49]
Morgan, R. A	1993	ATP/MgCl2	Did not test	NA	Did not test	NA	NA	NA	NA	Muscle and nerve stimulation	*In-vivo*	No mention	[50]
Pekoglu, E	2020	Trans-cinnamaldehyde (TCA)	Tested -Inhibited in treatment group	Neutrophil infiltration-MPO analysis-non-significant	Did not test	NA	NA	NA	NA	EDL	Ex-vivo	150	[29]
Quinones-Baldrich, W. J.	1991	Controlled reperfusion	Did not test	NA	Did not test	NA	NA	NA	NA	TA	In-situ		[46]
Ricles, L. M.	2016	MSCs + PEGylated fibrin gel	Did not test	NA	Did not test	NA	NA	NA	NA	Lateral gastrocnemius	In-situ	150	[39]
Rybalko, V.	2015	In vitro polarized M1 macrophages	Did not test	NA	Did not test	NA	NA	NA	NA	Gastrocnemius muscle	In-situ	150	[43]
Rybalko, V.	2017	mMPs	Did not test	NA	Did not test	NA	NA	NA	NA	Calf muscles	In-situ	150	[44]
Stahl, D.	2012	L-arginine at Warm I/R	Did not test	NA	Did not test	NA	NA	NA	NA	EDL	Ex-vivo		[30]
Wang	2021	Biological (NC-Exosomes)	Tested -Inhibited in treatment group	Inflammasome pathway(NLRP3, caspase 1, IL-1β, and IL-18)	Tested- Inhibited in treatment groups	NA	NA	NA	NA	Grip test-(Motor nerve test)-hind limb muscles	*In-vivo*	NA	[45]
Wilson, J. S.	2003	Dichloroacetate (DCA)	Did not test	NA	Did not test	NA	NA	NA	NA	Gastrocnemius muscle	*In-vivo*	150	[31]

Route of administration, and duration of treatment used in each study are listed in Table 1. The route of administration for these interventions were intravenous infusions (n = 4) [23, 24, 37, 38], intravenous (n = 9) [26, 28, 31, 35, 36, 41, 46, 48, 49], intramuscular (n = 8) [25, 33, 34, 39, 42–45], intraperitoneal (n = 1) [40], intraperitoneal infusion (n = 1) [32], local application (n = 1) [51], intra-arterially one-time bolus plus infusion (n = 1) [47], and gavage (n = 2) [27, 29]. Duration of treatment ranged from 30 mins to 6.5 hrs in eight studies [23, 24, 26, 32, 37, 38, 46, 50], in 15 studies a single bout of treatment was used [25, 28, 31, 33–36, 39, 41–45, 48, 51], one study used a one-time bolus followed by 60 mins of infusion [47], one-time treatment was used every day for 7 days [40] or for 5 days [27], three times treatment with two hour intervals [49] or 8 hour interval [29] was used, and the drug was given in 0.5 ml increments over 5 min interval for 1 hour [30] (**Table 1**).

The experimental endpoints varied considerably across studies (**Table 1**). The majority of studies terminated their experiments within 28 hours [24–26, 28–32, 35–38, 46–48, 50]. Only one study out of 29 terminated after 40 days [51]. Three studies terminated the experiments after 28 days [23, 34, 45] and another study terminated at 21 days [44]. Two studies terminated the experiments after 16 days [41, 42]. Two studies terminated the experiments at 14 days [33] and one of the studies terminated at two different time points, seven and 14 days [39]. One study terminated the experiments after 7 days [40]. One study terminated the experiments after five days [27]. One study terminated the experiments after two days [49].

#### Inflammation and edema

Out of the 29 studies, 11 reported inflammatory results (**Table 2**). Out of the 11 studies which reported inflammatory results, one study reported the inhibition of inflammation based on histological analysis of inflammatory cells such as, neutrophils, lymphocytes, and macrophages [23], one study reported the drug effects on inflammation based on microscopic observations (no details on how these observations were made are reported) [24], two studies reported inhibition of inflammation based on histology analysis of neutrophil infiltration alone [25, 36], and of the remaining six studies, two reported results based on CD68 immunoreactivity indicative for macrophage infiltration [33, 48], two studies from the same group [26, 27] and one other study [29] employed myeloperoxidase activity as a marker of skeletal muscle neutrophil infiltration to study inflammation, and the remaining study analyzed stained tissue for cellular inflammation with no specifics on the type of inflammatory cells [35], and one study analyzed inflammasome pathway genes (NLRP3, caspase 1, IL-1β, and IL-18) to study the drug effects on inflammation [45] (**Table 2**).

Edema was evaluated in only six studies [23, 26, 27, 35, 36, 47], while the remaining 23 studies did not test edema. Of these six studies, four calculated wet-to-dry ratios as an index of edema formation [23, 26, 27, 35], one used both histological analysis and calculated wet-to-dry ratio as an index of edema formation [36], one used amount of volume the muscle weight displaced and calculated the percentage weight change in the experimental muscle as a measurement of edema [47] (**Table 2**). All six studies tested intervention effects on edema reported a decrease in edema compared to their respective controls (**Table 2**).

#### Muscle function analysis

In line with the inclusion criteria, all 29 studies evaluated neuromuscular function in hind limb muscles (i.e., isometric force or torque either in tibialis anterior [TA]/gastrocnemius/ extensor digitorum longus [EDL]/soleus muscle [n = 28] or via grip test [n = 1]). Of these studies, eight tested isometric torque of the TA muscle [23, 26, 33, 46–49, 51], one tested isometric torque of TA and gastrocnemius muscle [37], five tested isometric torque of an EDL muscle [24, 25, 29, 30, 32], one tested isometric torque of EDL and soleus muscles [40], eight tested isometric torque of the gastrocnemius muscle [27, 28, 31, 35–37, 39, 44], one analyzed the calf muscles (gastrocnemius, soleus, and plantaris) [44], two studies from the same group tested isometric torque of the anterior crural muscles [41, 42], and one study analyzed neuromuscular function by directly stimulating muscle or nerve alone for function analysis of the effected limb [50]. The remaining 1 study analyzed neuromuscular function of hind limb muscles (grip test) [45]. For muscle function analysis, nine studies used *in-vivo* muscle tests [23, 31, 34, 41, 42, 45, 50, 51], seven used *ex-vivo* muscle function tests [24, 25, 28–30, 32, 40], and 13 used *in-situ* muscle function tests [26, 27, 33, 35–39, 43, 44, 46, 48, 49] (**Table 2**).

#### Study quality

**Fig 2:**
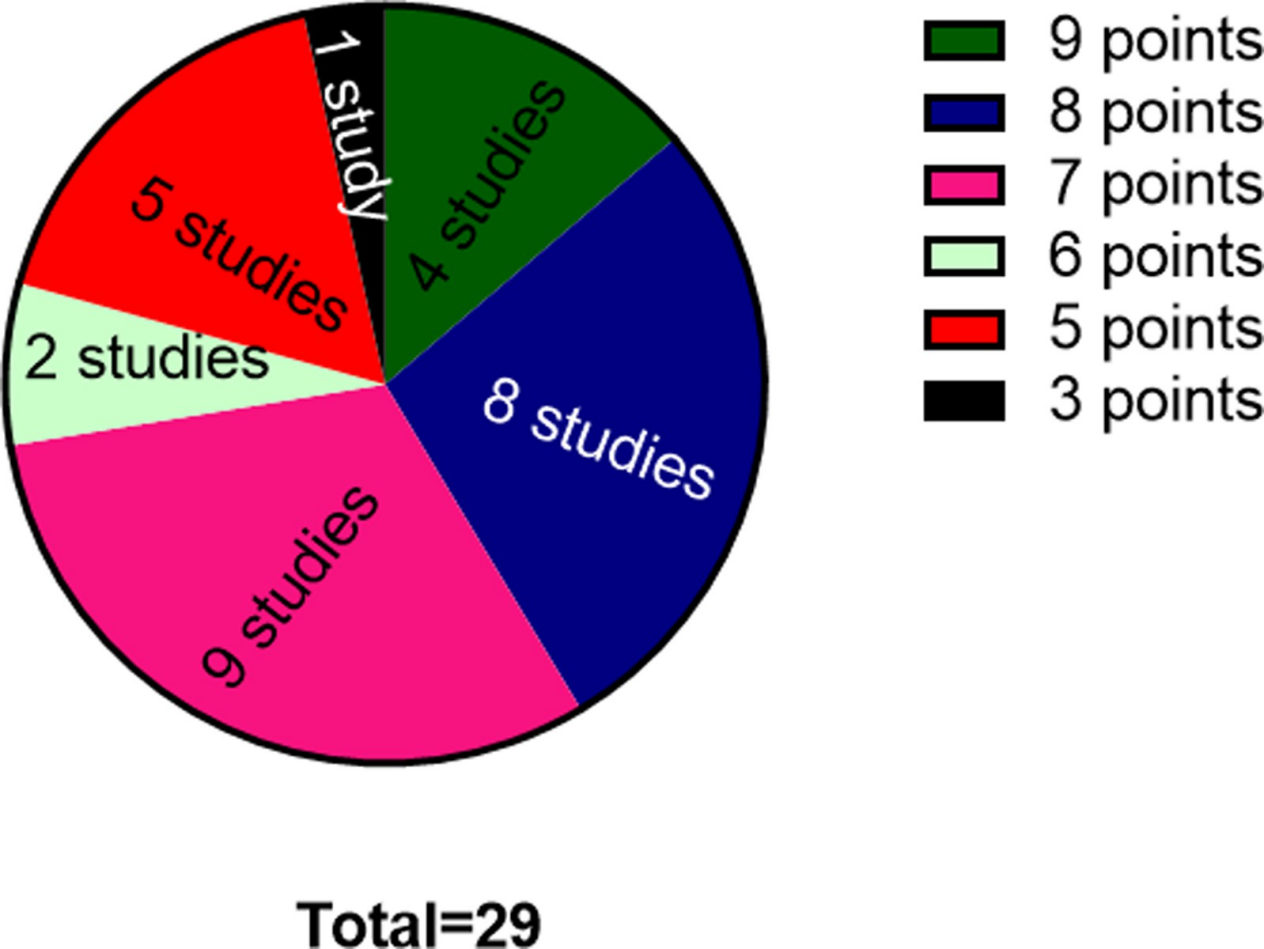
Analysis of study quality considering the quality assessment of studies included in systematic review as per the Systematic Review Centre for Laboratory Animal Experimentation’s (SYRCLE).

All the included studies were peer reviewed publications, free of selective outcome reporting, and all reported a description of the animals used. None of the studies, however, reported a sample size calculation. Out of 29 studies, 26 studies [23, 25–30, 32–47, 49–51] reported control of temperature and lighting for animal housing except for three studies [24, 31, 48]. 18 studies [23–30, 32, 33, 36–38, 40, 42, 45, 49, 51] reported that they performed random allocation of animals to control and treatment groups. 23 studies [23–30, 32, 33, 36–42, 44, 45, 47–49, 51] reported the timing of induction of disease and whether this was done after or before randomization. One study mentioned random selection of animals for outcome assessment [33]. Five studies reported a blinded outcome assessment [23, 32, 36, 48, 49], and the remaining studies did not use blinded outcome assessment. Five studies reported use of an anesthetics without significant effects on skeletal muscle injury [26–28, 39, 48]. 26 studies reported a compliance with animal welfare regulations [23, 25–30, 32–47, 49–51] and 11 studies mentioned a potential conflict of interest [23, 26–29, 34, 40, 41, 44, 45, 51]. The standard quality score included 29 studies that ranged from three to nine. Of which, four studies scored nine points [23, 26–28], eight studies scored eight points [29, 32, 33, 36, 40, 44, 45, 49, 51], nine studies scored seven points [25, 30, 34, 37–39, 41, 42], two studies scored six points [47, 48], five studies scored five points [24, 35, 43, 46, 50], and one study scored three points [31] (**Fig 2**). The methodological qualities of each study are summarized in **Table 3**.

**Table 3:** Methodological qualities of each study and their scores.

First Author	Year of publication	A	B	C	D	E	F	G	H	I	J	K	L	SCORE
Aurora, A.	2018	1	1	1	1	0	1	0	1	0	1	1	1	**9**
Bagis, Z.	2018	1	1	1	1	0	1	0	1	0	1	1	0	**8**
Chen, L. E.	1998	1	0	1	1	0	0	0	1	0	1	0	0	**5**
Chen, L. E.	1996	1	1	1	1	0	0	0	1	0	1	1	0	**7**
Chen, X. K	2011	1	1	1	1	1	0	0	1	0	1	1	0	**8**
Corona, B. T.	2014	1	1	0	1	0	0	0	1	0	1	1	1	**7**
Corona, B. T.	2013	1	1	1	1	0	0	0	1	0	1	1	0	**7**
Demirel, M	2013	1	1	1	1	0	0	0	1	0	1	1	1	**8**
Dillon, J. P.	2008	1	1	1	1	0	0	1	1	0	1	1	1	**9**
Dillon, J. P.	2006	1	1	1	1	0	0	1	1	0	1	1	1	**9**
Dyer, S. E.	2022	1	1	0	0	1	0	0	1	0	1	1	1	**7**
Feller, A. M	1989	1	0	0	1	0	1	1	1	0	1	0	0	**6**
Frey, S. P.	2019	1	1	1	1	0	0	0	1	0	1	1	1	**8**
Hirose, J.	1997	1	1	0	0	0	0	0	1	0	1	1	0	**5**
Hirose, J.	2001	1	1	1	1	0	1	0	1	0	1	1	0	**8**
Hoch, J. R	1991	1	1	0	1	0	0	0	1	0	1	1	0	**6**
Ikebe, K.	2001	1	1	1	1	0	0	0	1	0	1	1	0	**7**
Ikebe, K.	2002	1	1	1	1	0	0	0	1	0	1	1	0	**7**
Kingston, R.	2005	1	1	1	1	0	0	1	1	0	1	1	1	**9**
Mohler, L. R.	1996	1	1	1	1	0	1	0	1	0	1	1	0	**8**
Morgan, R. A	1993	1	1	0	0	0	0	0	1	0	1	1	0	**5**
Pekoglu, E	2020	1	1	1	1	0	0	0	1	0	1	1	1	**8**
Quinones-Baldrich, W. J.	1991	1	1	0	0	0	0	0	1	0	1	1	0	**5**
Ricles, L. M.	2016	1	1	0	1	0	0	1	1	0	1	1	0	**7**
Rybalko, V.	2015	1	1	0	0	0	0	0	1	0	1	1	0	**5**
Rybalko, V.	2017	1	1	0	1	0	0	0	1	0	1	1	1	**8**
Stahl, D.	2012	1	1	1	1	0	0	0	1	0	1	1	0	**7**
Wang	2021	1	1	1	1	0	0	0	1	0	1	1	1	**8**
Wilson, J. S.	2003	1	0	0	0	0	0	0	1	0	1	0	0	**3**

Note. Items rated as yes scored 1, and items rated as no or unable to determine or unsure both scored 0. Lower scores indicate a higher risk of bias.

A Peer reviewed publication

B Control of temperature and lighting (animal housing)

C Random allocation of animals to treatment or control

D Timing of disease induction before/after randomization (ischemia/trauma)

E Random selection of animals for outcome assessment

F Blind outcome assessment

G Use of anesthetic without significant effects on the skeletal muscle injury

H Animal model description (young, sex, weight)

I Sample size calculation

J Study free of selective outcome reporting (If all the pre-specified primary and secondary outcomes reported)

K Compliance with animal welfare regulations

L Statement of potential conflict of interests

### 3. Reported effects of interventions

#### Inflammation

8 studies separately interrogated interventions which used dexamethasone [25], muscle progenitor cells [33], hypertonic saline [26], pravastatin [27], EPC-K1 (L-ascorbic acid 2-[3,4-dihydro-2,5,7,8-tetramethyl-2-(4,8,12-trimethyltridecyl)-2H-1-benzopyran-6-yl hydrogen phosphate] potassium salt) radical scavenger [35, 36], trans-cinnamaldehyde [29], and exosomes [45] reported a reduction in inflammation when compared with their respective control groups. Interventions with FWB did not exacerbate the inflammation induced by I/R. S-nitroso-N-acetylcysteine, superoxide dismutase (SOD), and dimethylsulfoxide (DMSO) showed no apparent effect on inflammation **Table 2**.

### Edema

Five studies, which interrogated hypertonic saline (P<0.01) [26], pravastatin (P< 0.04) [27], EPC-K1 radical scavenger (tested in two independent studies; P = 0.01, and P = 0.003) [35, 36], SOD (P<0.04) [47] and mannitol (P<0.003) [47], showed a reduction in edema relative to their respective control groups. One of the studies, however, showed treatment with hextend exacerbated edema while the FWB treated group did not have any effect on edema when compared with a control group (I/R injured -untreated) [23] **Table 2.**

### Muscle isometric torque

In general, functional outcome measures improved after treatment in 22 of the included studies. All studies that explored chemical interventions showed an improvement in maximum isometric torque measurements, except for iloprost-(IL) [32], U74006F [49], L-NMMA + S-nitroso glutathione [38] and L-arginine [30]. 12 studies that experimented with the following agents: SNAC (100 nmol/min—116.8%; SNAC at 1 μmol/min—46.7441%; SNAC at 5 μmol/min—34.0218%; P<0.01 and P<0.001) [24], dexamethasone (512.36%, P<0.001) [25], L-carnitine (75.1418%, P<0.01) [40], hypertonic saline (7.5%) [26], EPC-K1 (tested in two independent studies, 143.902% and 22.0796%, p≤0.02) [35], L-NMMA (28.8462% and 143.526%, P<0.05) [36], taurine (taurine proximally-600.405% and proximally and distally-325.506%, P<0.005) [28], ATP/MgCl2 (after one hour Ischemia Nerve-2.24359%, four hours ischemia-Muscle 1000%, three hours after reperfusion- Muscle-2075% and Nerve-2350% increase, P<0.01) [50], TCA (75.1977%; P<0.05) [29], and DCA (30%, P<0.05) [31], reported an increase in maximum isometric torque when compared with their respective controls (**Table 4**). Three studies used both chemical and biological agents to test their effects on improving muscle contractile function [47, 48]. One of the studies interrogated SOD (5 hrs-69.7023%, P<0.005), and DMSO (at 5 hours-27.6708%; 8 hours-112.593%, P<0.01) individually [48] and the other study experimented both SOD and mannitol individually [47] and analyzed neuromuscular contractile force at two time points (3 hours and 20 hours). The study reported that SOD showed a decrease (P<0.002) in direct muscle stimulated contractile function at 3 hours, with no difference in nerve evoked contractile function (43.75%, ns), and mannitol at the three hour time point did not show a difference in both muscle and nerve contractile function, whereas at the 20 hour time point mannitol showed an increase (143.75%, P<0.05) in nerve stimulated contractile function (**Table 4**). The other study investigated sodium percarbonate (two doses) and catalase (fixed dose) at three different time points. Low dose of sodium percarbonate (week-1-260%, week-2- 234% and week-3-78.7%, P<0.005), showed an increase in muscle contractile function, whereas its high dose did not show an improvement on muscle function [34]. Out of eight studies that investigated biological interventions, two studies that used muscle progenitor cells-infected with lentiviruses [33] and Lin-BMCs [42], did not show an improved contractile muscle function upon analysis. Seven studies that interrogated biological interventions, FWB (2.86%, P = 0.02) [23], Lin-BMCs (20.2%, P<0.05) [41], CYR61 (58.96%, p = 0.0015) [51], bone marrow-derived mesenchymal stem cells (MSCs) in PEGylated fibrin hydrogel (14 days-54.6%, P<0.0125) [39], LPS/IFN-γ polarized mouse bone marrow macrophages (22.3%, P<0.05) [39], mMPs (IFN-gamma polarized mouse bone marrow macrophages) (mouse adipose stem cells (ASCs) in PBS-15.9% [44], polarized macrophages with adipose stem cells in PBS-25.43% [44], showed an improvement in maximum isometric torque of the muscle and NC-exosomes (109% increase compared to PBS; 30.078% Increase compared to Si-Exo), showed an improvement in neuromuscular strength [45]. One of the two studies that used the physical means of intervention, such as pump controlled reperfusion, did not show a statistical difference in any of the four groups tested [46] and the remaining study that used cold I/R reported a significant increase in maximum isometric torque when compared with the warm I/R group (P<0.01) [30].

**Table 4:** Muscle isometric torque.

				Isometric Torque					
First Author	Year of publication	Intervention name	Type of therapeutic agent	Control	Treated	% difference	Significance	Isometric Torque tested at Hz	Ref
Aurora, A.	2018	FWB- (Group- TK and HEM with FWB resuscitation)	Biological	TK 0.7+/- 0.03	FWB: 0.72+/-0.031; HEM:0.6+/-0.02	FWB: 2.86% increase; HEM: 95.6% decrease	FWB- P = 0.02	150	[23]
Bagis, Z.	2018	IL- iloprost	Synthetic chemical	8.508±1.313 N/cm2	IR24SF: 1.77±0.145 N/cm2; IR24IL: 3.025±0.562 N/cm2; IR48SF: 0.167±0.058 N/cm2; IR48IL:0.571±0.159 N/cm2	Decrease-IR24SF: = 79.1961%; IR24IL:-64.4452%; IR48SF:98.0371%; IR48IL: 93.3004%	P<0.001	150	[32]
LONG-EN CHEN	2018	S-nitroso-N-acetylcysteine (SNAC)	Synthetic chemical	41.832+/-8.168	I/R plus SNAC at 100 nmol/min -90.718+/-1.609; I/R plus SNAC at 1 μmol/min -61.386+/-6.064; I/R plus SNAC at 5 μmol/min -56.064+/-3.837	Increase- I/R plus SNAC at 100 nmol/min– 116.8%; I/R plus SNAC at 1 μmol/min– 46.7441%; I/R plus SNAC at 5 μmol/min– 34.0218%	P<0.01; P<0.001	120	[24]
Chen, L. E.	1996	Dexamethasone	Synthetic chemical	1.78+/-0.45	10.9+/-2.1	increase-512.36%	P<0.001	100	[25]
Chen, X. K	2011	Muscle progenitor cells- infected with lentiviruses	Biological	4.32+/-0.65 N/cm2	3.27+/-0.82 N/cm2	Decrease- 24.3056%	P<0.345 (not different between MPC and Veh)	150	[33]
Corona, B. T.	2014	LinBMCs	Biological	1.24+/-0.19 Nmm	1.49+/-0.11 Nmm	Increase-20.1613%	P<0.05	200	[41]
Corona, B. T.	2013	Lin-BMCs	Biological	1.36 +/- 0.08 Nmm	1.25+/- 0.11 Nmm	Decrease-8.08824%	P<0.05		[42]
Demirel, M	2013	L-carnitine	Chemical	2.82+/- 1.09 EDL N/cm2; 10.85+/-2.35 SOL N/cm^*2*^	4.939+/- 1.588 EDL N/cm2; 4.9+/-1.53 SOL N/cm^*2*^	Increase-75.1418%	P< .01	150	[40]
Dillon, J. P.	2008	Hypertonic saline (7.5% NaCl)	Chemical	2.13+/-0.55	2.69+/-0.46	Increase-26.2911%	P<0.03	50	[26]
Dillon, J. P.	2006	Pravastatin	Chemical	1.91+/-0.47	2.6+/-0.72	36.1257% increase	P<0.004	50	[27]
Dyer, S.E.	2022	Sodium percarbonate and catalase	Biological and chemical	Week1-12.2 ± 4.0; Week2-20.4±6.9; Week4-50.2± 7.8 Nmm/Kg	Week1-Low dose-43.9 ± 9.7; High Dose-19.8 ± 10.7; Week2- Low dose-68.2±10.6, High Dose-31.4 ± 13.0; Week4- Low dose- 9.7 ± 9.3, High Dose- 57.5 ± 15.5Nmm/Kg	Week1-Low dose- 260%, High dose-62.3%; Week2-Low dose-234.3%; High dose-53.9%; Week4-Low dose- 78.7%; High dose-14.5%	Week1-Low dose- P<0.005; Week2-Low dose- P<0.005; Week4-Low dose- P<0.005.	150	[34]
Feller, A. M	1989	SOD & DMSO	Biological and chemical	5 hrs- 57.1+/10.7-(%); 8 hrs- 27+/-10.1(%)	SOD-5 hrs-96.9+/-2.1; 8 hrs- 16.1+/-9.3; DMSO-5 hrs- 72.9+/-7.8(%); 8 hrs- 57.4+/-8.7(%)	SOD-5 hrs-69.7023% increase-; 8 hrs- 40.3704% decrease; DMSO—5 hrs-27.6708% increase; 8 hrs-112.593% increase	SOD-5hr-P<0.005; DMSO-8 hr-P<0.01	No mention	[48]
Frey, S. P.	2019	CYR61	Biological	1.73 +/- 0.90	2.75 +/- 0.50	58.9595% increase	P = 0.0015	No mention	[51]
Hirose, J.	1997	L-ascorbic acid 2-[3,4-dihydro-2,5,7,8-tetrameth- ~1-2-(4,8,12-trimethyltridecy1)-2H- 1 -benzopyran-6- yl hydrogen phosphate] potassium salt (EPC-K1)	Synthetic chemical	4.1+/-3.1	10.0 +/-2.3	143.902% increase	P≤0.02	200	[35]
Hirose, J.	2001	L-ascorbic acid 2-[3,4-dihydro-2,5,7,8-tetramethyl-2-(4,8,12- trimethyltridecyl)-2H-1-benzopyran-6-yl hydrogen phosphate] potassium salt (EPC-K1)	Chemical	7.79+/-1.41	9.51+/-0.98	22.0796% increase	P<0.02	200Hz	[36]
Hoch, J. R	1991	Superoxide dismutase and mannitol	Biological and chemical	3 hrs- Muscle stimulated:8.1+/-2.8; Nerve stimulated:8.0+/-3.0] [20 hrs-Muscle stimulated:3.7+/-1.0; Nerve stimulated:8.0+/-2.0]	[**Superoxide dismutase**—3 hrs- Muscle stimulated:5.3+/-1.9; Nerve stimulated:11.5+/-5.4][20 hrs-Muscle stimulated:3.3+/-1.4; Nerve stimulated:7.3+/-3.5] [**Mannitol**—3 hrs dta: Muscle stimulated:10.3+/-2.1; Nerve stimulated:12.3+/-3.7][20 hrs dta: Muscle stimulated:15.4+/-3.3; Nerve stimulated:19.5+/-4.9]	[**Superoxide dismutase**—3 hrs- Muscle stimulated:34.5679% decrease; Nerve stimulated:43.75% increase][20 hrs-Muscle stimulated:10.8108% decrease; Nerve stimulated:8.75% decrease] [**Mannitol**—3 hrs data: Muscle stimulated:27.1605% increase ; Nerve stimulated:53.75% increase][20 hrs data: Muscle stimulated:316.216% increase; Nerve stimulated: 143.75% increase]	Muscle stimulated-SOD- 3 hrs- P<0.002; Nerve stimulated-mannitol-20 hrs-P<0.05	Reference provided	[47]
Ikebe, K.	2001	L-NMMA	Chemical	TA-2.6 (0.09); Gastrocnemius muscle-	TA-3.35 (0.13); Gastrocnemius muscle-	28.8462% increase	P<0.05	150	[37]
Ikebe, K.	2002	L-NMMA; L-NMMA + S-nitrosoglutathione; SOD	Chemical	3.63 (0.35)	L-NMMA: 8.84 (1.23); L-NMMA + S-nitrosoglutathione:3.31 (0.39); SOD: 7.44 (0.77)	NMMA-143.526% increase; NMMA+S-nitrosoglutathione-8.81543% decrease; SOD-104.96% increase	P<0.05	200	[38]
Kingston, R.	2005	Taurine	Chemical	9.88+/-11.52	Taurine-Proximally 69.2+/- 55.37; Taurine-distally; 74.78+/- 50.44 Taurine-Proxi+dist 42.04+/- 39.49	Taurine proximally-600.405% increase; Taurine distally- 656.883% increase; Taurine-proxim_Dis-325.506% increase	Taurine proximally-P = 0.013; Taurine distally-P<0.091; Taurine-proxim_Dis-P<0.005	No mention	[28]
Mohler, L. R.	1996	U74006F (tirilazad mesylate, 21-[4-(2,6-di-1-pyrrolidinyl-4-pyrimidinyl)-1-piperazinyl]16m-methyl-pregna-1,4,9(11)-triene-3,20 dione methane-sulfonate	Chemical	**2 hrs–**Nerve stimulation:1340 +/- 236; Direct: 1631 +/- 80–**4 Hrs** Direct stimulation:1321 +/- 132	**2 hrs–**Nerve stimulation:1320+/-295; Direct: 1469+/-99–**4 Hrs** Direct stimulation:1389+/-84	2 hrs–Nerve stimulation: 1.49254% decrease; Direct: 9.62687% increase; 4 Hrs Direct stimulation-3.65672% increase	No statistically significant difference between the groups is seen	100	[49]
Morgan, R. A	1993	ATP/MgCl2	Chemical	Muscle stimulation 0- immediately prior to arterial clamping Mean (SE) 895 (26) Nerve stimulation 0- immediately prior to arterial clamping Mean (SE) 842 (28); Muscle stimulation 1 hr after ischemia Mean (SE) 448 (71) Nerve stimulation 1 hr after ischemia Mean (SE) 312 (52); Muscle stimulation 4 hr after ischemia Mean (SE) 0 (0) Nerve stimulation 4 hr after ischemia Mean (SE) 1 (1); Muscle stimulation 3 hr after reperfusion Mean (SE) 4 (3) Nerve stimulation 3 hr after reperfusion Mean (SE) 2 (2)	Muscle stimulation 0- immediately prior to arterial clamping Mean (SE) 892 (52) Nerve stimulation 0- immediately prior to arterial clamping Mean (SE) 813 (65); Muscle stimulation 1 hr after ischemia Mean (SE) 439 (72) Nerve stimulation 1 hr after ischemia Mean (SE) 319 (61); Muscle stimulation 4 hr after ischemia Mean (SE) 1 (1) Nerve stimulation 4 hr after ischemia Mean (SE) 0 (0); Muscle stimulation 3 hr of reperfusion Mean (SE) 87 (19) Nerve stimulation 3 hr of reperfusion Mean (SE) 49 (10);	0 hr- Muscle- 0.335196% decrease Nerve-3.44418% decrease: 1 hr Isch-Muscle- 2.00893% decrease Nerve-2.24359% increase; 4 hr isch-Muscle 1000% increase- Nerve-1000% decrease; 3 hr after reperfusion- Muscle2075% increase- Nerve-2350% increase	Both muscle and nerve stimulation-P<0.01	No mention	[50]
Pekoglu, E	2020	trans-cinnamaldehyde (TCA)	Synthetic chemical	307.31 ± 217.8	538.40 ± 355.8	75.1977% increase	P<0.05	150	[29]
Quinones-Baldrich, W. J.	1991	Controlled reperfusion	Pump Controlled reperfusion	Normal reperfusion-0.574 -/+ 0.073;	Controlled reperfusion-0.552+- 0.057; Leukopenic/thrombocytopenic Controlled reperfusion-0.618 +/- 0.151; Fibrinolytic reperfusion-0.482 +/- 0.066	Controlled reperfusion- 3.83275% decrease; Leukopenic/thrombocytopenic Controlled reperfusion- 7.66551% increase; Fibrinolytic reperfusion-16.0279% decrease	No statistically significant difference between the four groups is seen		[46]
Ricles, L. M.	2016	MSCs + PEGylated fibrin gel	Biological	7 days: 72.02 +/- 3.05; 14 days: 59.03 ± 8.69%	[MSCs + PEGylated fibrin gel: 7 days- 76.85 +/- 20.49;14 days- 91.26 ± 4.06%] [MSCs in FBS: 7 days- 81.08 +/- 9.76; 14 days -71.74 ± 9.21%] [PEGylated fibrin gel: 7 days-70.15 +/- 5.12; 14 days- 68.12 ± 6.23%]	[MSCs + PEGylated fibrin gel: 7 days-6.70647% increase; 14 days-54.5994% increase [MSCs in FBS: 7 days-12.5798% increase; 14 days -21.5314% increase] [PEGylated fibrin gel: 7 days- 2.5965% decrease; 14 days-15.3989% increase]	MSCs + PEGylated fibrin gel-14 days-P<0.0125	150	[39]
Rybalko, V.	2015	In vitro polarized M1macrophages	Biological	13.23 +/- 0.43	[In vitro polarized M1macrophages: 16.18 +/- 0.42] [M0 macrophages: 11.55+/- 0.53]	[In vitro polarized M1macrophages: 22.2978% increase] [M0 macrophages: 12.6984% decrease]	P<0.05	150	[43]
Rybalko, V.	2017	mMPs	Biological	14.04+/-1.16	[IFN-gamma polarized mouse bone marrow macrophages: 12.15+/-1.08] [mouse adipose stem cells (ASCs) in PBS: 16.27+/-1.67] [polarized macrophages with adipose stem cells in PBS:17.61+/-2.64]	[IFN-gamma polarized mouse bone marrow macrophages: 13.4615% decrease][mouse adipose stem cells (ASCs) in PBS: 15.8832% increase] polarized macrophages with adipose stem cells in PBS:25.4274% increase	P<0.05	150	[44]
Stahl, D.	2012	L-arginine at Warm I/R	Temperature effects	Maximum contractile force 74.3+12	[Warm I/R: Maximum contractile force 39.7+10 Cold/IR: Maximum contractile force 80.5+9; L-arginine at warm I/R: Maximum contractile force 61.4+11;	[Warm I/R: Maximum contractile force 46.568% decrease; Cold/IR: Maximum contractile force 8.34455% increase; L-arginine at warm I/R: Maximum contractile force 17.362% decrease;	Warm I/Rgroups showed a statistically significant decrease in contractileforce when compared with the sham, cold I/R, andL-argininegroups (P,0.05). Cold I/R group was not significantlydifferent from the sham and/or theL-arginine group but foundto be significantly increased compared with the warm I/Rgroup (P,0.01).L-Arginine, similarly, was not significantly different in the average contractile force generated comparedwith the sham and/or the cold I/R group. However, theL-arginine group showed significantly improved average contractile forces compared with the warm I/R group (P,0.05).	10	[30]
Wang	2021	Biological (NC-Exosomes)	Biological	28 days- Si-Exo-192.7+/-5.71; PBS-131.9+/-8.6 (g)	28 days-NC-Exo-275.6+/-7.9	109% increase compared to PBS; 30.078% Increase compared to Si-Exo.	PNC-Exo compared to PBS-P = 0.0001; NC-Exo compared to Si-Exo-P = 0.0001	NA	[45]
Wilson, J. S.	2003	Dichloroacetate (DCA)	Chemical	2.0 +/- 0.6 mins	2.6+/-0.3 min	30% increase	P<0.05	150	[31]

Effect size calculation of each study revealed that it had different effect size with sample size. Out of 29 studies, 15 studies, which showed large effect sizes on muscle function improvement are SNAC at 100 nmol/min (3.3807, 95% C.I. 1.9371 to 4.8242) 1 μmol/min (1.0412, 95% C.I. -0.0031 to 2.0855), 5 μmol/min (0.8475, 95% C.I. -0.2112 to 1.9062) [24], dexamethasone (2.2698, 95% C.I. 1.0133 to 3.5264) [25], hypertonic saline (1.1045, 95% C.I. 0.1126 to 2.0964) [26], pravastatin (1.1349, 95% C.I. 0.1394 to 2.1304) [27], low dose of sodium per carbonate (Week 1 is 1.9363, 95% C.I. 0.7699 to 3.1027; Week 2 is 2.0654, 95% C.I. is 0.8756 to 3.2552) [34], SOD at five hours (1.9314, 95% C.I. 0.7447 to 3.1181) [48], DMSO at eight hours (1.2686, 95% C.I. 0.1105 to 2.4267) [48], CYR61 (1.34, 95% C.I. 0.3346 to 2.3455) [51], EPC-K1 (2.1616, 95% C.I. 0.9282 to3.395) [35], Mannitol at 20 hours (Muscle stimulated: 1.959, 95% C.I. 0.6846 to 3.2334; Nerve evoked: 1.2545, 95% C.I. 0.1085 to 2.4006) [47], L-NMMA (TA: 2.0226, 95% C.I. 1.0389 to 3.0063; Gastrocnemius muscle: 1.0002, 95% C.I. 0.1515 to 1.8489) [37], L-NMMA in another study showed (2.5767, 95% C.I. 1.0459 to 4.1074) [38], ATP/MgCl2 (Nerve stimulation- 3 hr after reperfusion: 2.6609, 95% C.I. 1.2225 to 4.0992) [50], MSCs plus PEGylated fibrin gel at 14 days (4.7521, 95% C.I. 2.3284 to 7.1757) [39], MSCs in FBS at both seven (1.253, 95% C.I. 0.1028 to 2.6088) [39], and 14 days (1.4195, 95% C.I. 0.0326 to 2.8065) [39], PEGylated fibrin gel at 14 days (1.2023, 95% C.I. -0.1447 to 2.5492) [39], M1 macrophages (2.9411, 95% C.I. 1.3704 to 4.5118) [44], and NC-exosomes (12.0275, 95% C.I. 6.6125 to 17.4425) [45] and DCA (1.2649, 95% C.I. 0.1914 to 2.3384) [31] (**Table 5**).

**Table 5:** Effect sizes of isometric torque.

General details			Isometric torque		Sample #	effect size		
First Author	Year of publication	Name of intervention	Control	Treated	n =	d	95% C.I.	Ref
Aurora, A.	2018	FWB-(Group- TK and HEM with FWB resuscitation)	TK 0.7+/- 0.03	FWB: 0.72+/-0.031; HEM:0.6+/-0.02	8	FWB: -0.2478; HEM: -1.7387	FWB: -1.2315 to 0.7359; HEM: -2.889 to -0.5883	[23]
Bagis, Z.	2018	IL- iloprost	8.508±1.313 N/cm2	IR24SF: 1.77±0.145 N/cm2; IR24IL: 3.025±0.562 N/cm2; IR48SF: 0.167±0.058 N/cm2; IR48IL:0.571±0.159 N/cm2	7	IR24SF: -7.2135; IR24IL: -5.4292; IR48SF: -8.9752; IR48IL: -8.4868	IR24SF:-10.0835 to -4.3436; IR24IL: -7.6967 to -3.1617; IR48SF: -12.4608 to -5.4896; IR48IL: -11.8003 to -5.1733	[32]
Chen, L. E.	1998	S-nitroso-N-acetylcysteine (SNAC)	41.832+/-8.168	I/R plus SNAC at 100 nmol/min: 90.718+/-1.609; I/R plus SNAC at 1 μmol/min: 61.386+/-6.064; I/R plus SNAC at 5 μmol/min: 56.064+/-3.837	C = 8; t = 10/8/7; for 100/1/5	100 nmol/min: 3.3807; 1 μmol/min: 1.0412; 5 μmol/min: 0.8475	100 nmol/min: 1.9371 to 4.8242; 1 μmol/min: -0.0031 to 2.0855; 5 μmol/min: -0.2112 to 1.9062	[24]
Chen, L. E.	1996	Dexamethasone	1.78+/-0.45	10.9+/-2.1	8	2.2698	1.0133 to 3.5264	[25]
Chen, X. K	2011	Muscle progenitor cells- infected with lentiviruses	4.32+/-0.65 N/cm2	3.27+/-0.82 N/cm2	6	-0.6346	C.I.: -1.7944 to 0.5251	[33]
Corona, B. T.	2014	Lin-BMCs	1.24+/-0.19 Nmm	1.49+/-0.11 Nmm	C = 8; T = 7	0.6071	C.I.: -0.4303 to 1.6445	[41]
Corona, B. T.	2013	Lin-BMCs	1.36 +/- 0.08 Nmm	1.25+/- 0.11 Nmm	C = 9; T = 10	-0.3847	C.I.: -1.2935 to 0.5241	[42]
Demirel, M	2013	L-carnitine	2.82+/- 1.09 EDL N/cm2; 10.85+/-2.35 SOL N/cm^*2*^	4.939+/- 1.588 EDL N/cm2; 4.9+/-1.53 SOL N/cm^*2*^	9	EDL: 0.5218; SOL: -1.0527	EDL: -0.3939 to 1.4375; SOL: -2.0135 to -0.092	[40]
Dillon, J. P.	2008	Hypertonic saline (7.5% NaCl)	2.13+/-0.55	2.69+/-0.46	9	1.1045	0.1126 to 2.0964	[26]
Dillon, J. P.	2006	Pravastatin	1.91+/-0.47	2.6+/-0.72	9	1.1349	0.1394 to 2.1304	[27]
Dyer, S.E.	2022	Sodium percarbonate and catalase	Week1-12.2 ± 4.0; Week2-20.4±6.9; Week4-50.2± 7.8 Nmm/Kg	Week1-Low dose-43.9 ± 9.7; High Dose-19.8 ± 10.7; Week2- Low dose-68.2±10.6, High Dose-31.4 ± 13.0; Week4- Low dose- 9.7 ± 9.3, High Dose- 57.5 ± 15.5Nmm/Kg	C = 12; LD = 6; HD = 6	Week1-Low dose: 1.9363; High Dose: 0.4389; Week2- Low dose: 2.0654, High Dose: 0.4403; Week4- Low dose: -1.66, High Dose: 0.2992	Week1-Low dose: 0.7699 to 3.1027; High Dose: -0.5515 to 1.4293; Week2- Low dose: 0.8756 to 3.2552, High Dose: -0.5502 to 1.4308; Week4- Low dose: -2.78 to -0.54, High Dose: -0.6856 to 1.2841	[34]
Feller, A. M	1989	SOD/DMSO	5 hrs- 57.1+/10.7-(%); 8 hrs- 27+/-10.1(%)	SOD-5 hrs-96.9+/-2.1; 8 hrs- 16.1+/-9.3; DMSO-5 hrs- 72.9+/-7.8(%); 8 hrs- 57.4+/-8.7(%)	All groups = 8; 8hrs = 6	SOD-5 hrs:1.9314; 8 hrs: -0.4462; DMSO-5 hrs: 0.6217; 8 hrs:1.2686	SOD-5 hrs: 0.7447 to 3.1181; 8 hrs: -1.5175 to 0.6251; DMSO-5 hrs: -0.3817 to 1.625; 8 hrs:0.1105 to 2.4267	[48]
Frey, S. P.	2019	CYR61	1.73 +/- 0.90	2.75 +/- 0.50	C = 11; T+8	1.34	0.3346 to 2.3455	[51]
Hirose, J.	1997	L-ascorbic acid 2-[3,4-dihydro-2,5,7,8-tetrameth- ~1-2-(4,8,12-trimethyltridecy1)-2H- 1 -benzopyran-6- yl hydrogen phosphate] potassium salt (EPC-K1)	4.1+/-3.1	10.0 +/-2.3	8	2.1616	0.9282 to3.395	[35]
Hirose, J.	2001	L-ascorbic acid 2-[3,4-dihydro-2,5,7,8-tetramethyl-2-(4,8,12- trimethyltridecyl)-2H-1-benzopyran-6-yl hydrogen phosphate] potassium salt (EPC-K1)	7.79+/-1.41	9.51+/-0.98	8	0.5354	EPC-K1: -0.462 to 1.5328	[36]
Hoch, J. R	1991	Superoxide dismutase and mannitol	3 hrs- Muscle stimulated:8.1+/-2.8; Nerve stimulated:8.0+/-3.0][20 hrs-Muscle stimulated:3.7+/-1.0; Nerve stimulated:8.0+/-2.0]	[**Superoxide dismutase**—3 hrs- Muscle stimulated:5.3+/-1.9; Nerve stimulated:11.5+/-5.4][20 hrs-Muscle stimulated:3.3+/-1.4; Nerve stimulated:7.3+/-3.5] [**Mannitol**—3 hrs dta: Muscle stimulated:10.3+/-2.1; Nerve stimulated:12.3+/-3.7][20 hrs dta: Muscle stimulated:15.4+/-3.3; Nerve stimulated:19.5+/-4.9]	7	[**Superoxide dismutase**—3 hrs- Muscle stimulated: -0.5119; Nerve stimulated: 0.3271][20 hrs- Muscle stimulated: -0.1408; Nerve stimulated: -0.1003] [**Mannitol**—3 hrs dta: Muscle stimulated: 0.3629; Nerve stimulated: 0.5333][20 hrs dta: Muscle stimulated: 1.959; Nerve stimulated: 1.2545]	[**Superoxide dismutase**—3 hrs- Muscle stimulated: -1.5765 to 0.5528; Nerve stimulated: -0.7275 to 1.3817] [20 hrs-Muscle stimulated: -1.1897 to 0.9081; Nerve stimulated: -1.1486 to 0.948] [**Mannitol**—3 hrs dta: Muscle stimulated: -0.6933 to 1.4191; Nerve stimulated: -0.5328 to 1.5994][20 hrs dta: Muscle stimulated: 0.6846 to 3.2334; Nerve stimulated: 0.1085 to 2.4006]	[47]
Ikebe, K.	2001	L-NMMA	TA-2.6 (0.09); Gastrocnemius muscle-0.77+/-0.151	TA-3.35 (0.13); Gastrocnemius muscle-1.6+/-0.32	12	TA: 2.0226; Gastroc: 1.0002	TA:1.0389 to 3.0063; Gastroc: 0.1515 to 1.8489	[37]
Ikebe, K.	2002	L-NMMA; L-NMMA + S-nitrosoglutathione; Super oxide dismutase (SOD)	3.63 (0.35)	L-NMMA: 8.84 (1.23); L-NMMA + S-nitrosoglutathione:3.31 (0.39); SOD: 7.44 (0.77)	6	L-NMMA: 2.5767; NMMA + S-nitrosoglutathione: -0.3862; SOD: 2.8489	NMMA: 1.0459 to 4.1074; NMMA + S-itrosoglutathione: -1.5283 to 0.7559; SOD:1.2428 to 4.455	[38]
Kingston, R.	2005	Taurine	9.88+/-11.52	Taurine-Proximally 69.2+/- 55.37; Taurine-distally; 74.78+/- 50.44 Taurine-Proxi+dist 42.04+/- 39.49	C = 4; T-P = 5; T-D = 6; T-P+D = 4	T test; No mention of SE/SD	T test; No mention of SE/SD	[28]
Mohler, L. R.	1996	U74006F (tirilazad mesylate, 21-[4-(2,6-di-1-pyrrolidinyl-4-pyrimidinyl)-1-piperazinyl]16m-methyl-pregna-1,4,9(11)-triene-3,20 dione methane-sulfonate	**2 hrs—**Nerve stimulation:1340 +/- 236; Direct: 1631 +/- 80–**4 Hrs** Direct stimulation:1321 +/- 132	**2 hrs—**Nerve stimulation:1320+/-295; Direct: 1469+/-99–**4 Hrs** Direct stimulation:1389+/-84	7	**2 hrs—**Nerve stimulation: -0.0301; Direct: -0.7348–**4 Hrs** Direct stimulation: 0.2509	**2 hrs—**Nerve stimulation: -1.0778 to 1.0179; Direct: -1.8172 to 0.3476–**4 Hrs** Direct stimulation: -0.8008 to 1.3027	[49]
Morgan, R. A	1993	ATP/MgCl2	Muscle stimulation 0- immediately prior to arterial clamping Mean (SE) 895 (26) Nerve stimulation 0- immediately prior to arterial clamping Mean (SE) 842 (28); Muscle stimulation 1 hr after ischemia Mean (SE) 448 (71) Nerve stimulation 1 hr after ischemia Mean (SE) 312 (52); Muscle stimulation 4 hr after ischemia Mean (SE) 0 (0) Nerve stimulation 4 hr after ischemia Mean (SE) 1 (1); Muscle stimulation 3 hr after reperfusion Mean (SE) 4 (3) Nerve stimulation 3 hr after reperfusion Mean (SE) 2 (2)	Muscle stimulation 0- immediately prior to arterial clamping Mean (SE) 892 (52) Nerve stimulation 0- immediately prior to arterial clamping Mean (SE) 813 (65); Muscle stimulation 1 hr after ischemia Mean (SE) 439 (72) Nerve stimulation 1 hr after ischemia Mean (SE) 319 (61); Muscle stimulation 4 hr after ischemia Mean (SE) 1 (1) Nerve stimulation 4 hr after ischemia Mean (SE) 0 (0); Muscle stimulation 3 hr of reperfusion Mean (SE) 87 (19) Nerve stimulation 3 hr of reperfusion Mean (SE) 49 (10);	7	Muscle stimulation 0- immediately prior to arterial clamping: -16.9864 Nerve stimulation 0- immediately prior to arterial clamping: -0.2366; Muscle stimulation 1 hr after ischemia: -0.0514 Nerve stimulation 1 hr after ischemia: 0.0504; Muscle stimulation 4 hr after ischemia:0.5774 Nerve stimulation 4 hr after ischemia: -0.5774; Muscle stimulation 3 hr of reperfusion: -0.5477 Nerve stimulation 3 hr of reperfusion: 2.6609	Muscle stimulation 0- immediately prior to arterial clamping: -23.3648 to -10.6081 Nerve stimulation 0- immediately prior to arterial clamping: -1.2879 to 0.8147; Muscle stimulation 1 hr after ischemia: -1.0992 to 0.9964 Nerve stimulation 1 hr after ischemia: -0.9974 to 1.0982; Muscle stimulation 4 hr after ischemia: -0.4919 to 1.6466 Nerve stimulation 4 hr after ischemia: -1.6466 to 0.4919; Muscle stimulation 3 hr of reperfusion: -1.6148 to 0.5194 Nerve stimulation 3 hr of reperfusion: 1.2225 to 4.0992	[50]
Pekoglu, E	2020	Trans-cinnamaldehyde (TCA)	307.31 ± 217.8	538.40 ± 355.8	8	0.7834	TCA: -0.2335 to 1.8003	[29]
Quinones-Baldrich, W. J.	1991	Controlled reperfusion	Normal reperfusion-0.574 -/+ 0.073;	Controlled reperfusion-0.552+- 0.057; Leukopenic/thrombocytopenic Controlled reperfusion-0.618 +/- 0.151; Fibrinolytic reperfusion-0.482 +/- 0.066	C = 10; CR = 8; L/T-R = 9; FR = 5	Controlled reperfusion: -0.1145; Leukopenic/thrombocytopenic Controlled reperfusion: 0.1319; Fibrinolytic reperfusion: -0.45	Controlled reperfusion: -1.0449 to 0.816; Leukopenic/thrombocytopenic Controlled reperfusion: -0.7696 to 1.0334; Fibrinolytic reperfusion: -1.3619 to 0.4618	[46]
Ricles, L. M.	2016	MSCs + PEGylated fibrin gel	7 days: 72.02 +/- 3.05; 14 days: 59.03 ± 8.69%	[MSCs + PEGylated fibrin gel: 7 days- 76.85 +/- 20.49;14 days- 91.26 ± 4.06%] [MSCs in FBS: 7 days- 81.08 +/- 9.76; 14 days -71.74 ± 9.21%] [PEGylated fibrin gel: 7 days-70.15 +/- 5.12; 14 days- 68.12 ± 6.23%]	5	[MSCs + PEGylated fibrin gel: 7 days: 0.3297;14 days: 4.7521] [MSCs in FBS: 7 days: 1.253; 14 days: 1.4195] [PEGylated fibrin gel: 7 days: -0.4438; 14 days: 1.2023]	[MSCs + PEGylated fibrin gel: 7 days: -0.9183 to 1.5777;14 days: 2.3284 to 7.1757] [MSCs in FBS: 7 days: -0.1028 to 2.6088; 14 days: 0.0326 to 2.8065] [PEGylated fibrin gel: 7 days: -1.6985 to 0.811; 14 days: -0.1447 to 2.5492]	[39]
Rybalko, V.	2015	In vitro polarized M1 macrophages	13.23 +/- 0.43	[In vitro polarized M1macrophages: 16.18 +/- 0.42][M0 macrophages: 11.55+/- 0.53]	C = 7; M1 = 6; M0 = 5	[In vitro polarized M1macrophages: 2.9411] [M0 macrophages: -1.5909	[In vitro polarized M1macrophages: 1.3704 to 4.5118] [M0 macrophages: -2.9033 to -0.2786]	[43]
Rybalko, V.	2017	mMPs	14.04+/-1.16	[IFN-gamma polarized mouse bone marrow macrophages: 12.15+/-1.08] [mouse adipose stem cells (ASCs) in PBS: 16.27+/-1.67] [polarized macrophages with adipose stem cells in PBS:17.61+/-2.64]	5 to 9/grp	No mention of how many animals each group used		[44]
Stahl, D.	2012	L-arginine at Warm I/R	Maximum contractile force 74.3+12	[Warm I/R: Maximum contractile force 39.7+10 Cold/IR: Maximum contractile force 80.5+9; L-arginine at warm I/R: Maximum contractile force 61.4+11;	5	[Warm I/R: Maximum contractile force: -1.5663 Cold/IR: Maximum contractile force: 0.2923; L-arginine at warm I/R: Maximum contractile force:-0.43	[Warm I/R: Maximum contractile force: -2.9832 to -0.1493 Cold/IR: Maximum contractile force: -0.9539 to 1.5385; L-arginine at warm I/R: Maximum contractile force: -1.6839 to 0.8238	[30]
Wang	2021	Biological (NC-Exosomes)	28 days- Si-Exo-192.7+/-5.71; PBS-131.9+/-8.6 (g)	28 days-NC-Exo-275.6+/-7.9	5	12.0275	6.6125 to 17.4425	[45]
Wilson, J. S.	2003	Dichloroacetate (DCA)	2.0 +/- 0.6 mins	2.6+/-0.3 min	8	1.2649	0.1914 to 2.3384	[31]

Four studies, which showed medium effect sizes are Lin-BMCs (0.6071, 95% C.I. -0.4303 to 1.6445) [41], L-carnitine (EDL, 0.5218, 95% C.I. 0.1126 to 2.0964) [40], EPC-K1 (0.5354, 95% C.I. -0.462 to 1.5328; 2001) [36], SOD at three hours (Muscle stimulated: -0.5119, 95% C.I. -1.5765 to 0.5528) [47], and mannitol at three hours (Nerve stimulated: 0.5333, 95% C.I. -0.5328 to 1.5994) [47]. ATP/MgCl2 at a different time point and experimental condition showed a medium effect size. (Muscle stimulation 4 hr after ischemia: 0.5774, 95% C.I. -0.4919 to 1.6466) [50]. All remaining studies showed small effect sizes [23, 30, 32, 33, 42, 46, 49]. L-NMMA alone showed a large effect size, whereas, L-NMMA plus S-nitrosoglutathione showed a small effect size [38]. Sodium bicarbonate tested at higher dose showed a small effect size [34]. SOD tested at eight hour time-point showed a small effect size [48]. The study which tested ATP/MgCl2 one hour after ischemia (by direct stimulation of either muscle or nerve alone), showed a small effect size [50]. In the same study muscle stimulation after 1 hour of reperfusion showed a small effect size [50]. MSCs plus PEGylated fibrin gel in combination or PEGylated fibrin gel alone at seven day time point showed a small effect size [39]. M0 macrophages showed a small effect size in one study [44].

## Discussion

### 1. Summary of the overall results

No clinical studies qualified for inclusion within this systematic review. Within the preclinical literature, the efficacy of the non-surgical interventions for ACS with respect to edema, inflammation, and muscle-contractile function was assessed in 29 studies. Although edema and inflammation are the key players in ACS pathology, not all of the included studies investigated the effects of interventions on these symptoms. 22 studies showed improvement in muscle contractile function after the treatments. The evidence available from the present study revealed that 12 chemical interventions (i.e., SNAC, dexamethasone, L-carnitine, hypertonic saline, EPC-K1, L-NMMA, taurine, ATP/MgCl2, TCA, DCA, DMSO, and mannitol) while seven biological interventions (i.e., FWB, Lin-BMCs, CYR61, bone marrow-derived mesenchymal stem cells (MSCs), LPS/IFN-γ polarized mouse bone marrow macrophages, mMPs, and exosomes) and three combinatorial interventions (i.e. chemical and biological co-therapies: SOD/DMSO/mannitol and Sodium percarbonate and catalase) were found to facilitate improvement in muscle contractile function. Likewise, One physical intervention (i.e. warmer reperfusion liquid (maintained at 34°C–36°C) was shown to mediate an improvement in muscle isometric torque after injury. Effect size calculations revealed that out of all the studies, the study which tested NC-exosomes expressing cPWWP2A repaired I/R injury by inhibiting Rb1- mediated NLRP3 inflammasome through the cPWWP2A/Rb1/AMPKα2/NLRP3 signaling pathway showed the largest effect on muscle function improvement. The remaining studies which showed large effect sizes on muscle function improvement are: SNAC, dexamethasone, hypertonic saline, pravastatin, low dose of sodium percarbonate, SOD (at five hours), DMSO (at eight hours), CYR61, EPC-K1, Mannitol (at 20 hours), L-NMMA, MSCs in FBS (at both seven and 14 days), PEGylated fibrin gel (at 14 days), M1 macrophages and DCA.

### 2. Limitations of the included studies

This review provides useful information with respect to the possibility of using above identified agents as non-surgical therapies to elicit improved functional outcomes following ACS. However, the articles included within this systematic review have the following limitations: 1) None of the studies provided information on sample size calculations. The adequate sample size is required for scientific rigor and appropriate interpretation of findings. 2) Only one study reported that animals were randomly selected for outcome assessments. Randomization is used in human studies, whereas in animal experiments, this is not widely adopted. 3) Out of 29 studies, only five provided blinded outcome assessment. The random selection of subjects and blinded outcome assessments have been shown to reduce bias in human clinical trials. Hence, the unduly biased pre-clinical studies may not be considered a part of the rationale for clinical trials. 4) Only five studies investigated the effects of anesthesia on skeletal muscle injury. Inhalant anesthetics have shown effects on neurotransmission pathways and calcium activation of muscle. It is important to study anesthetic effects on muscle injury. There are speculations that inhalant anesthetics may directly involve the contractile proteins actin or myosin. Thus, having control animals to study anesthesia effects alone is recommended to account for these variables. 5) Only 11 studies included the statement “disclosure of potential conflict” out of 29 studies. Declaring conflicts of interest is critical for maintaining the integrity of unbiased professional assessment of the publications. Previously, the inclusion of this statement was largely neglected in reputed journals, but presently, it is necessary to report conflicts of interest statements before acceptance for publication. 6) The majority of included studies, except for three, reported random allocation of animals to treatment or control groups. It is well known that failure to randomize will lead to the overestimation of treatment benefits of interventions across outcome measures. 7) Out of 29 studies, six did not specify the timing of ischemia/trauma induction before/after randomization, making the analysis difficult. As discussed earlier, the randomization of animals plays an important role in outcome assessment. Further, only five studies adopted female animals, and it cannot be ignored that both genders will be developing ACS due to trauma with a different pattern of sensitivity towards trauma. Healing patterns may also differ in these genders due to differences in the type of sexual hormones. Another limitation is that none of the studies used a crushed/trauma model to create ischemia for testing intervention efficacy on muscle function. As discussed previously, immediately after ACS is diagnosed, the fascia should be cut open within 3–6 hrs of the injury to prevent irreversible damage. This point should be considered when designing the experiments. Another limitation is that three of the included studies terminated the study within 1 or 2 hrs, which is before the onset of ACS. Early termination of studies made it difficult to derive/understand results that are meaningful to apply for treating trauma-related ACS. Although these models represent the pathophysiological mechanism that causes ACS with increased pressure and ischemia leading to tissue damage, clearly, all of the models reviewed here are not representative of the exact pathophysiology of ACS present in the trauma patient population, where severe destruction of soft tissue with/without fractures, localized reductions in regional blood flow to traumatized tissue and trauma induced inflammatory milieu are seen.

As discussed earlier, high-quality animal studies are crucial for the translation of animal data into clinical studies [52]. Therefore, we suggest that while designing experiments/analyzing results, authors should try to avoid the limitations listed above and follow standard guidelines for animal studies to improve the accuracy of the experimental data [53].

### 3. Implications

As previously discussed, fasciotomy is the current standard of care for ACS and the only clinical option for immediate relief of the increased intra-compartmental pressure. Part of the sequelae of ACS and its subsequent release via fasciotomy is a reperfusion injury which carries with it a multi-week timeline over which muscle function is slowly recovered. Given the prevalence of ACS in military trauma, this recovery timeline is a significant burden to our wounded Service members and ultimately the readiness of the joint Forces. As such identifying and gaining knowledge of the effective treatments for reperfusion injury is paramount to designing adjunct, non-surgical treatment strategies to fasciotomy for ACS. Thus, given the lack of bonafide ACS studies in the literature, studies focused on preventing or ameliorating the pathophysiology of I/R injury were used to gain insights into how various readily available interventions might help reduce ACS symptoms and improve muscle function. The majority of the studies evaluated here terminated the experiments at/after >4 hrs except for three studies [30, 31, 47]. Biological interventions used individually are the most effective in protecting the tissue from I/R damage and improved muscle contractile function. These interventions tested muscle contractile function after a prolonged time (7 to 40 days after intervention) compared to other treatments, which analyzed this parameter within 24 hrs. Despite the relevance of studies that investigated the intervention effects on I/R damage within 24 hours of injury, they are limited in understanding the effects of interventions that may prolong the ischemic time that tissue can resist before the tissue damage occurs. An increase in the length of the ischemic period will lead to an increase in cell death with irreversible damage followed by loss of structural integrity of the affected tissue. Therefore, specifically targeted therapies are necessary to activate cell survival programs to overcome the pathologic events associated with ACS.

From the reviewed literature, it is apparent that inflammation plays a significant role in creating ACS conditions [54]. Compounds that possess anti-inflammatory properties may show improved efficacy in reducing ACS, importantly when improving muscle contractile function in the affected limb. A majority of the included studies showed improvement in muscle function whereas, only 10 included studies reported/analyzed effects on inflammation. Out of 10 studies, eight studies showed inhibitory effects on inflammation after treatments. Similarly, edema is the result of acute inflammation from an injury [54]. Edema results in an increased tissue pressure, metabolic insults due to tissue necrosis and increased infiltration of inflammatory cells, inducing defective endogenous muscle recovery mechanisms. Thus, the development of therapeutic strategies is required to minimize the pathological inflammatory processes and direct towards tissue formation. Among the included studies, only a few aimed to analyze intervention effects on edema. Currently, this review was able to show that the majority of interventions tested in the included studies rescued the muscle from pathological conditions of ACS with an improvement in muscle contractile function. The agents discussed in this review may have the potential to rescue the muscle from pathological conditions of ACS developed by trauma.

From this paper, we have observed a lack of therapies which directly target skeletal muscle regeneration as a means to accelerate functional recovery after ACS. Given the critical role that myofiber damage and necrosis plays in the pathology of ACS, it seems that such interventions represent an underexplored therapeutic option which warrants further investigation as a means to accelerate functional recovery of the afflicted. To this end interventions (e.g. small molecules or direct cellular therapies) which might promote an expanded pool of activated satellite cells in the affected compartment would be of particular interest either as a monotherapy or in conjunction with a number of compounds targeting inflammation, oxidative stress, or nitric oxide metabolism as reviewed herein.

## Conclusions

Based on the findings presented herein, both chemical and biological interventions may improve muscle-contractile function following I/R within an ACS conditions. However, caution should be exercised in the interpretation of the data as variability in experimental designs, analysis measures, and limited analysis of edema and inflammation limits our understanding of how these interventions compare to each other. In addition, there are paucity of studies available on the use of extremity trauma-mediated ACS models which utilized muscle contractile force as an (primary) outcome measure. Therefore, further work is warranted to evaluate the efficacy of non-surgical interventions on functional outcomes in ACS that is caused by extremity trauma. Further research in this direction will help wounded soldiers who develop ACS to have shorter recovery times and restoration of full muscle function.

## Supporting information

S1 Checklist PRIMSA abstract checklist. (DOCX)

S2 Checklist PRISMA 2020 main checklist. (DOCX)
